# Dietary inflammatory index and type 2 diabetes in US women: a cross-sectional analysis of the National Health and Nutrition Examination Survey, 2007–2018

**DOI:** 10.3389/fnut.2024.1455521

**Published:** 2024-08-14

**Authors:** Tingyan Mo, Man Wei, Jinyan Fu

**Affiliations:** ^1^Nutrition Section, Women’s Health Department, Changning Maternity and Infant Health Hospital, East China Normal University, Shanghai, China; ^2^Department of Laboratory Medicine, Gansu Provincial Hospital of Traditional Chinese Medicine, Lanzhou, China

**Keywords:** dietary inflammatory index, type 2 diabetes, NHANES, cross-sectional study, obesity, oral health

## Abstract

**Objective:**

Type 2 diabetes (T2D) is a major public health concern in the United States and worldwide. The dietary inflammatory index (DII) is a useful tool for assessing dietary inflammation. Although much research links the DII to diabetes, little is known about the relationship in adult women with a reproductive history in the United States. We aimed to investigate how the relationship between the DII and T2D varies among different subgroups of American women.

**Methods:**

Secondary data from the National Health and Nutrition Examination Survey from 2007 to 2018 were analyzed. Cross-sectional analysis of 8,394 American women aged 20 years or older who had at least one live birth. The main outcome was the diagnosis of T2D. Multivariate survey-weighted regression was used to determine the odds ratio (OR) and 95% confidence interval (95%CI) for the association between DII and T2D. A weighted restricted cubic spline (RCS) model was constructed to establish OR curves at three knots to examine the dose–response association between DII and T2D. Additionally, a weighted subgroup analysis was performed in a fully adjusted model to verify that the association was robust.

**Results:**

The study main found a significant association between the DII and T2D (OR = 1.19, 95%CI: 1.12, 1.26, *p <* 0.001). Participants in the highest third of DII scores had a 56% increased risk of T2D (OR = 1.56, 95%CI: 1.16, 2.10; *p* for trend = 0.003) compared with those in the lowest third of DII scores, after adjusting for all covariates. The multivariable RCS demonstrated a linear association between DII and T2D (*p* = 0.892). The subsidiary found that subgroup analyses revealed a significant variation in the association between DII and T2D according to obesity, oral health, and poverty-income ratio (PIR) status. Among non-obese women, the OR was 1.22 (95%CI: 1.08, 1.37); among women with good oral health, the OR was 1.17 (95%CI: 1.07, 1.28); among women with low PIR, the OR was 1.17 (95%CI: 1.05, 1.30); and among women with high PIR, the OR was 1.26 (95% CI: 1.07, 1.48).

**Conclusion:**

Our findings suggest that there is a significant association between DII and T2D and that oral health, obesity, and PIR status may influence the relationship between DII and T2D risk. Further studies are warranted to validate our results and evaluate whether the results are similar in other populations.

## 1 Introduction

Type 2 diabetes (T2D) is the most common form of diabetes and a significant cause of death and health issues. It also imposes a heavy and rapidly increasing burden on the United States economy (1). The International Diabetes Federation states that over 536 million people between the ages of 20 and 70 years worldwide have diabetes, resulting in a global health spending of around USD 673 billion in each year. T2D is now a major public health problem (2).

Recent studies suggest that inflammation is a key factor in the development of diabetes (3). People with diabetes have higher levels of inflammatory cytokines such as tumor necrosis factor-α and interleukin 6 (4, 5). The persistent expression of pro-inflammatory proteins, has been observed even in the presence of controlled blood glucose levels. Furthermore, the increasing association between inflammation and end-stage diabetes and its associated complications, including in the female population, has been widely discussed in the scientific literature.

Notably, diet plays a crucial role in preventing and managing diabetes (6). Healthy diets that include plenty of vegetables, whole grains, and fruits are associated with lower levels of inflammation (7). On the other hand, high-calorie Western diets combined with an unhealthy lifestyle can lead to chronic metabolic inflammation (8).

The DII is the definitive index of a food’s inflammatory potential. It is derived from a comprehensive analysis of food parameters. Work to develop the dietary inflammatory index (DII) began in 2004 (9), leading to an improved scoring system in 2014 (10), which can measure the inflammatory impact of different dietary patterns (11). A higher positive DII score indicates a more pro-inflammatory diet, while a lower negative DII score indicates an anti-inflammatory effect of the diet.

The well-being of women is of paramount importance to society. Their future quality of life is inextricably linked to their current state of health and wellbeing. Furthermore, women bear the crucial responsibility of nurturing the next generation. It is therefore vital that they are in good health. To date, no relationship between DII and T2D has been reported in women with a history of childbearing. In this study, using data from the National Health and Nutrition Examination Survey (NHANES)from the period 2007–2018, we aimed to investigate the connection between dietary inflammation potential and T2D in women in the United States.

## 2 Materials and methods

### 2.1 Data source and study sample

The NHANES is a nationwide survey conducted by the National Centre for Health Statistics in the United States. Its primary objective is to assess the health and nutritional status of the non-institutionalized population in the United States (12). NHANES has a cross-sectional design and uses a stratified, multistage probability sampling design for data collection, which is conducted every 2 years. The NHANES protocol is approved by the National Center for Health Statistics Research Ethics Review Board, and all participants provide written informed consent. No additional Institutional Review Board approval was required for secondary analysis (13). NHANES data are available on the NHANES website^1^.

Data from this cross-sectional study were gathered from the NHANES database over six different time spans from 2007 to 2018, totaling 69,943 participants at the outset.

**Figure 1:**
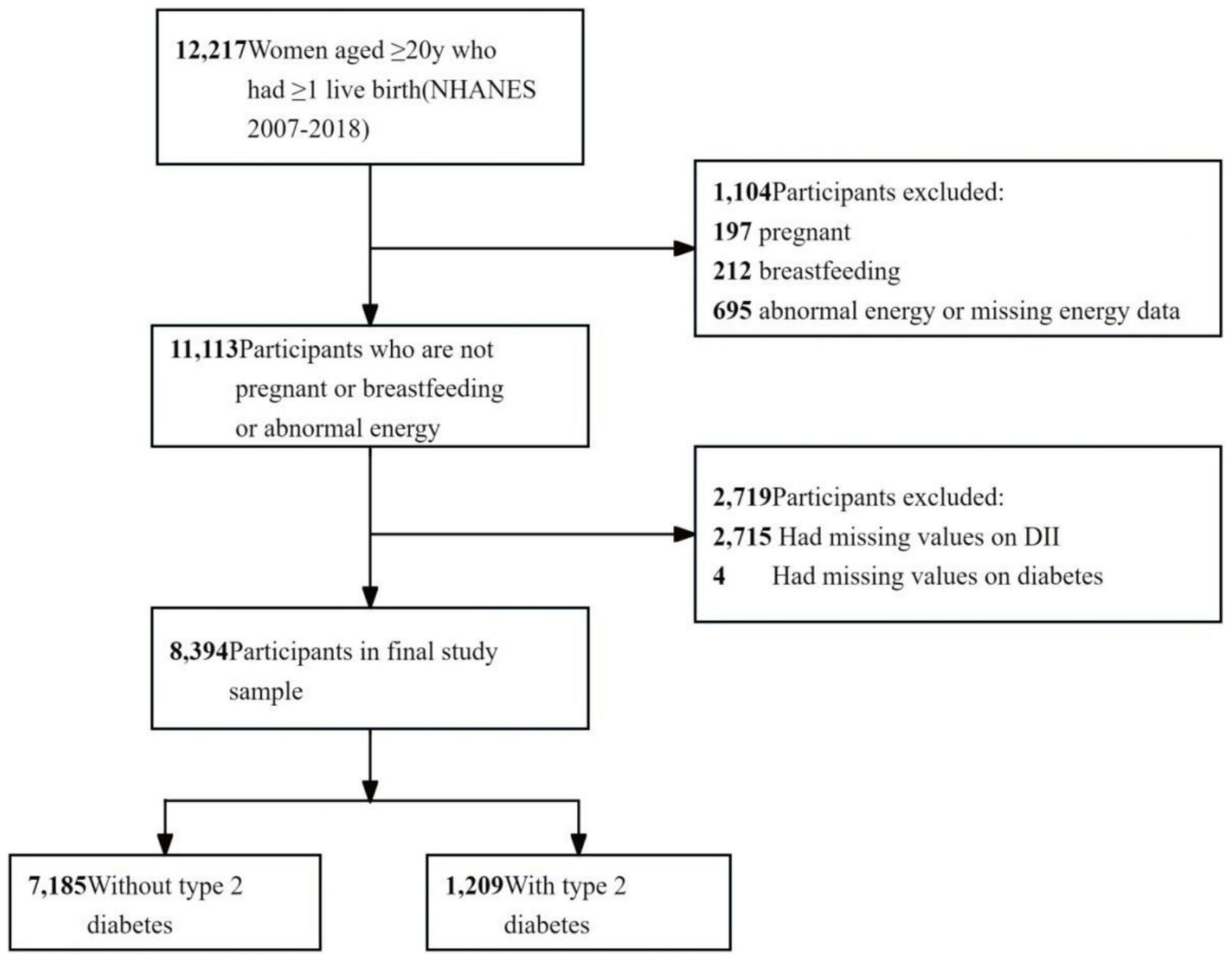
Flowchart of the sample selection.

The study included female individuals who met the following criteria: (1) age ≥ 20 years, (2) having at least one live birth, (3) not pregnant or breastfeeding, (4) no abnormal energy (total energy intake of 500 to 5,000 kcal/day) (14) or missing energy data, and (5) complete 24-h dietary interviews and diabetes mellitus data. A flowchart of participant enrollment is shown in Figure 1.

### 2.2 Exposure variable

The DII was designed as the exposure variable. The DII is now a widely recognized parameter for assessing overall dietary inflammation. Its structural validity and calculation methodology have been published (15). Dietary intake was documented and validated by the first 24-h dietary recall interview in this study, and we calculated DII scores based on the 24-h dietary data (16, 17). First, we calculated various dietary parameters and their respective Z-scores for each participant. The values were then converted into median percentiles, and a standardized overall inflammatory impact score was calculated for each median percentile, considering several dietary factors. By summing the DII scores for each participant, we obtained an “overall DII score” that reflected the individual’s dietary inflammatory potential. The dietary parameters included in this study covered a wide range of factors, including alcohol; protein; fiber; β-carotene; cholesterol; carbohydrates; energy; fats; n-3, n-6, polyunsaturated, monounsaturated, and saturated fatty acids; thiamin; magnesium; zinc; selenium; iron; riboflavin; folic acid; vitamins A, B-6, B-12, C, D, and E; caffeine; and niacin (16, 18). This comprehensive approach allowed us to assess the relationship between dietary inflammatory potential and the prevalence of T2D.

### 2.3 Outcome variable

The outcome of interest was the development of T2D, based on self-report questionnaires administered before the physical examination at home, using the computer-assisted personal interviewing (interviewer-administered) system, defined as a woman having been told by a doctor or health professional that she had diabetes (19).

### 2.4 Covariates

We included the following demographic and socioeconomic factors: age, college education (yes/no), marital status (married or living with a partner, living alone), poverty income ratio (PIR, <1.3, 1.3–3.5, >3.5), body mass index (BMI, < 30, ≥30 kg/m^2^), white blood cell count (10^9^/L), family history of diabetes (yes/no), parity (1 or 2, and ≥ 3 children), and health insurance (yes/no) (20). Race and ethnicity were categorized as non-Hispanic White and “other” (non-Hispanic Black, Hispanic, Mexican American, other Hispanic, and multiracial) (21, 22). Tobacco use was assessed through the following question: “Have you smoked at least 100 cigarettes in your lifetime?” (23). Alcohol use was assessed through the question, “Have you had at least 12 drinks of alcohol in 1 year?” (24). Physical activity (PA) was measured using a self-administered questionnaire and calculated as minutes of metabolic equivalents per week (MET-min/week). MET-min/week = MET × weekly frequency × duration of each PA. If PA = 0, participants did not engage in any PA. Otherwise, they had constant or intermittent PA. Subsequently, PA was classified into two groups based on the American PA guidelines. Active PA was defined as more than 599 MET, or more than 149 min of moderate PA, or more than 74 min of vigorous PA (25). History of gestational diabetes was obtained by asking: “During pregnancy, were you ever told by a doctor or other health professional that you had diabetes, sugar diabetes, or gestational diabetes?” Oral health was assessed using a series of self-reported questionnaires asking participants to rate the condition of their teeth and gums as poor, fair, good, very good, or excellent (20). In this analysis, overall oral health status was coded as a binary variable. We recorded good, very good, or excellent as “good” (and assigned a value of 1) and recorded fair or poor as “poor” (and assigned a value of 0) (26).

### 2.5 Statistical analysis

Our analysis followed the NHANES guidelines for statistical analysis, taking into account complex sampling designs and weights. We used dietary weights for weighted analysis, specifically the dietary day-one sample weight for the NHANES 2007–2018 data (21). The sampling weights for 2007–2018 were calculated as 1/6 × dietary day-one sample weight. All percentages were survey-weighted to be generalizable to the non-institutionalized population of women in the US.

The DII was analyzed according to NHANES database guidelines to estimate T2D. Continuous variables were presented as mean ± standard deviation, whereas categorical variables were expressed as percentages. A multivariate imputation method for missing data from iterative imputation was implemented using a Bayesian Ridge model as the estimator at each step of the round-robin imputation, which was initially transformed into three tertiles. To calculate the *p* values for the basic characteristics of the analyzed individuals with categorical variables, we used the chi-square test. For continuous variables, we used the Kruskal–Wallis rank-sum test to compute the *p* values. Three weighted models were used to represent the hierarchical adjustment for the regression models. Model I was unadjusted; Model II was adjusted for age, race, college education, marital status, and PIR; and Model III was adjusted for age, race, college education, marital status, PIR, PA, family history of diabetes, history of gestational diabetes, parity, BMI, and oral health. Furthermore, the relationship between the DII score and T2D was analyzed using RCS in the fully adjusted model, treating the DII score as a continuous variable. Interaction and subgroup analyses were performed using logistic regression models based on age, race, college education level, marital status, PIR, PA, gestational diabetes history, family history of diabetes, and parity. We further investigated the relationship between DII and T2D in the non-obese, good-oral health, low-PIR, and high-PIR populations. DII was included in the analyses successively as a continuous variable and as a tertile variable. To evaluate the robustness of our results, we conducted sensitivity analyses by changing the outcome variable to fasting glucose level.

Statistical power estimates were not performed because the sample size was determined based solely on the provided data. Statistical analyses were performed using R software (version 4.2.1; R 12 Foundation for Statistical Computing, Vienna, Austria), the R survey package (version 4.1**–**1), and Free Statistics software (version 1.9.2; Beijing Free Clinical Medical Technology Co, Ltd) 0.21 All tests were two-sided, and a significance level of *p* < 0.05 was used.

## 3 Results

### 3.1 Study population

The dataset included 12,217 women aged more than 20 years who had at least one live birth. Of these, 409 women were excluded for pregnancy or breastfeeding, 695 for abnormal energy intake, 2,715 for missing dietary data, and 4 for missing diabetes data. The final group comprised 8,394 participants (weighted *n* = 59,647,927). Further details are provided in Figure 1 and Supplementary Table S1.

### 3.2 Baseline characteristics

Table 1 presents the characteristics of the study participants according to their DII tertiles. This study included 8,394 individuals with a mean age of 53.72 years. The DII scores ranged from −4.94 to 4.74, with a mean of 1.56. Participants with higher DII scores tended to have lower education levels, lower income ratios, higher physical activity levels, and higher BMI and white blood cell. Tobacco use increased as DII increased from Q1 to Q3; conversely, alcohol consumption and oral health decreased.

**Table 1:** Characteristics of survey participants included in analysis (*N* = 8,394), National Health and Nutrition Examination Survey, 2007–2018.

Charcateristic	Dietary inflammatory index (DII)^C^				
	Overall	Q1	Q2	Q3	*P* value
	*n* = 8,394	*n* = 2,798	*n* = 2,798	*n* = 2,798	
**Type 2 diabetes (%)**					
No	7185.00 (85.60)	2483.00 (88.74)	2387.00 (85.31)	2315.00 (82.74)	<0.0001
Yes	1209.00 (14.40)	315.00 (11.26)	411.00 (14.69)	483.00 (17.26)	
Age, mean ± SD (years)	53.72 (16.17)	54.72 (15.38)	52.88 (16.30)	53.57 (16.77)	0.0001
**Race/ethnicity (%)**					
Non-Hispanic White	3677.00 (43.81)	1336.00 (47.75)	1173.00 (41.92)	1168.00 (41.74)	<0.0001
Others^a^	4717.00 (56.19)	1462.00 (52.25)	1625.00 (58.08)	1630.00 (58.26)	
**College education (%)**					
No	4175.00 (49.79)	1035.00 (37.02)	1460.00 (52.25)	1680.00 (60.09)	<0.0001
Yes	4211.00 (50.21)	1761.00 (62.98)	1334.00 (47.75)	1116.00 (39.91)	
**Marital status (%)**					
Married or living with a partner	4821.00 (57.46)	1736.00 (62.09)	1620.00 (57.92)	1465.00 (52.38)	<0.0001
Living alone	3569.00 (42.54)	1060.00 (37.91)	1177.00 (42.08)	1332.00 (47.62)	
Poverty income ratio, median, IQR	1.92 [1.04, 3.77]	2.55 [1.28, 4.81]	1.89 [1.05, 3.67]	1.49 [0.88, 2.87]	<0.0001
**Tobacco use (%)**					
No	5148.00 (61.35)	1857.00 (66.39)	1760.00 (62.95)	1531.00 (54.72)	<0.0001
Yes	3243.00 (38.65)	940.00 (33.61)	1036.00 (37.05)	1267.00 (45.28)	
**Alcohol use (%)**					
No	3479.00 (41.49)	1058.00 (37.84)	1157.00 (41.40)	1264.00 (45.22)	<0.0001
Yes	4907.00 (58.51)	1738.00 (62.16)	1638.00 (58.60)	1531.00 (54.78)	
Physical activity, mean ± SD (MET)	2241.48 (4474.22)	2248.53 (3933.59)	2030.93 (4255.02)	2444.98 (5138.69)	0.004
**History of gestational diabetes (%)**					
No	7726.00 (92.21)	2574.00 (92.23)	2576.00 (92.16)	2576.00 (92.23)	0.9948
Yes	653.00 (7.79)	217.00 (7.77)	219.00 (7.84)	217.00 (7.77)	
**Family history of diabetes (%)**					
No	4508.00 (54.60)	1603.00 (58.14)	1457.00 (53.00)	1448.00 (52.65)	<0.0001
Yes	3748.00 (45.40)	1154.00 (41.86)	1292.00 (47.00)	1302.00 (47.35)	
**Parity**^**b**^ **(%)**					
1 or 2	4371.00 (52.07)	1581.00 (56.50)	1471.00 (52.57)	1319.00 (47.14)	<0.0001
≥3	4023.00 (47.93)	1217.00 (43.50)	1327.00 (47.43)	1479.00 (52.86)	
BMI, mean ± SD (kg/m^2^)	30.12 (7.39)	29.25 (7.17)	30.31 (7.29)	30.81 (7.63)	<0.0001
**Oral health (%)**					
Poor	2860.00 (35.25)	799.00 (29.10)	985.00 (36.54)	1076.00 (40.27)	<0.0001
Good	5254.00 (64.75)	1947.00 (70.90)	1711.00 (63.46)	1596.00 (59.73)	
WBC, mean ± SD (10^9^/L)	7.25 (2.30)	7.00 (2.24)	7.32 (2.23)	7.43 (2.41)	<0.0001
**Health insurance (%)**					
No	1545.00 (18.41)	450.00 (16.08)	514.00 (18.37)	581.00 (20.76)	<0.0001
Yes	6849.00 (81.59)	2348.00 (83.92)	2284.00 (81.63)	2217.00 (79.24)	

DII, Dietary Inflammator Index. MET, metabolic equivalent. BMI, body mass index. WBC, white blood cell. ^a^“Other” includes Mexican American, Other Hispanic, Non-Hispanic Black, Other Race - Including Multi- Racial. ^b^Number of children. ^c^Data are presented as unweighted numbers (unweighted percentage) for categorical variables and mean (SD) for continuous variables.

Out of all participants, 1,209 persons had T2D, accounting for 14.40% of the total participants. The basic characteristics of the excluded and included participants are presented in Supplementary Table S1. Supplementary Table S2 presents the participants’ results of weighted analyses.

### 3.3 Association between DII and T2D

The correlation between DII and the risk of T2D was analyzed using weighted multiple logistic regression. Higher DII scores were associated with an increased risk of T2D (Table 2). In Model I, the OR was 1.19 (95%CI: 1.12, 1.26, *p* < 0.001); in Model II, the OR was 1.14 (95%CI: 1.08, 1.22, *p* < 0.001); and in Model III, the OR was 1.12 (95%CI: 1.05, 1.21, *p* = 0.002). The relationship between DII and T2D was significant in all models, with the DII as a continuous variable. The unadjusted model (Model I) showed that each one-unit increase in DII score raised the risk of T2D by 19%, whereas in the fully adjusted model (Model III), this risk increased by 12%.

**Table 2:** Weighted ORs (95%CIs) of the association between DII and type 2 diabetes.

Variables	Model I		Model II		Model III	
	OR (95%Cl)	*P* value	OR (95%Cl)	*P* value	OR (95%Cl)	*P* value
**DII**	1.19 (1.12, 1.26)	<0.001	1.14 (1.08, 1.22)	<0.001	1.12 (1.05, 1.21)	0.002
**DII**						
Q1	Reference		Reference		Reference	
Q2	1.32 (1.03, 1.68)	0.029	1.25 (0.96, 1.62)	0.091	1.17 (0.86, 1.61)	0.314
Q3	1.87 (1.46, 2.40)	<0.001	1.62 (1.25, 2.10)	<0.001	1.56 (1.16, 2.10)	0.004
*P* for trend		<0.001		<0.001		0.003

Model I: Crude model. Model II: Adjust for age, race, college education, marital status, poverty income ratio. Model III: Adjust for age, race, college education, marital status, poverty income ratio, physical activity, family history of diabetes, history of gestational diabetes, parity, BMI, oral health. DII, Dietary Inflammatory Index. OR, odds ratio. CI, confidence interval. BMI, body mass index.

The statistical significance of this relationship remained even after dividing the DII into three parts. DII scores ranged from 2.44 to 4.74 in the top tertile, from 1 to 2.44 in the middle tertile, and from −4.94 to 1 in the bottom tertile. In Model I, individuals with the highest DII scores in the top tertile had an 87% increased risk of T2D (OR = 1.87, 95%CI: 1.46, 2.40; *p* for trend<0.001) compared with those in the bottom tertile of DII scores. This risk increased by 56% (OR = 1.56, 95%CI: 1.16, 2.10; *p* for trend = 0.003) after adjusting for all covariates in Model III.

The dose–response relationship between DII and T2D was assessed using the RCS. The results indicated a linear relationship between the DII and T2D (*p* = 0.892) (Supplementary Figure S1).

### 3.4 Subgroup and sensitivity analyses

**Figure 2:**
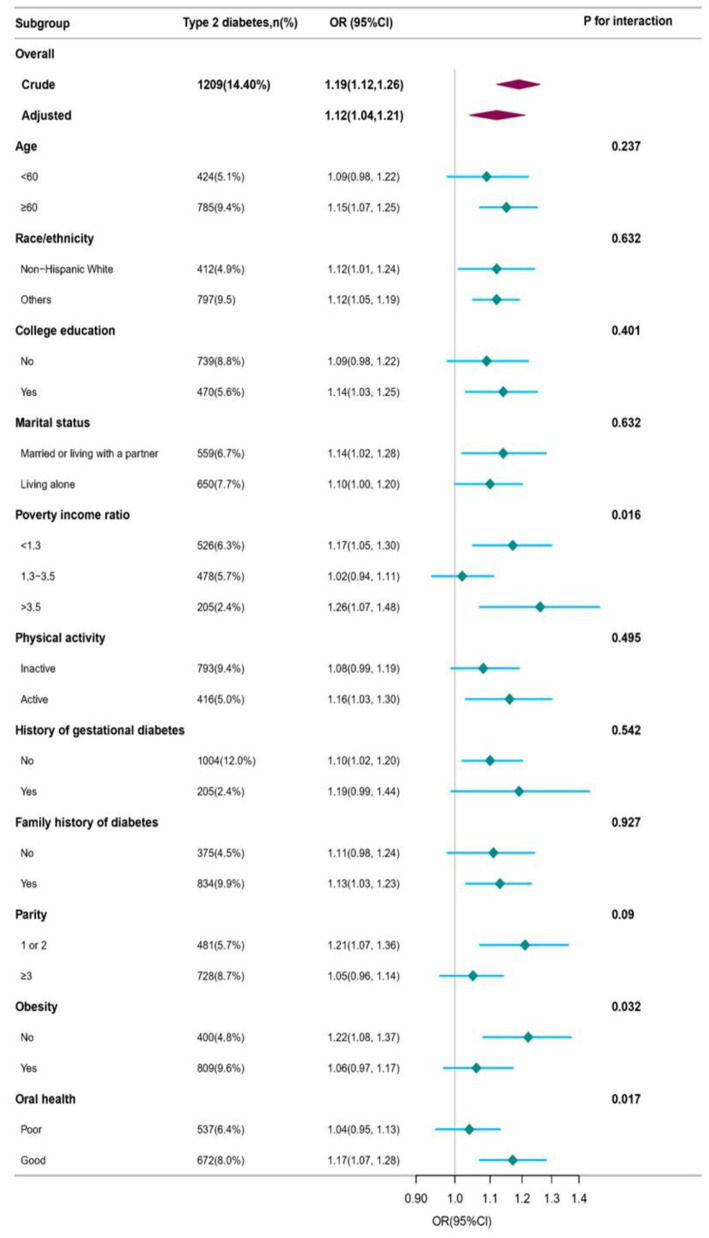
Association between dietary inflammatory index and type 2 diabetes.

Weighted subgroup analyses were performed using a completely adjusted model to investigate the association between DII and T2D. The results showed that the DII score had a significant positive association with T2D in most subgroups. However, no significant associations were found in the subgroups with age < 60 years, no college education, nophysical activity, history of gestational diabetes, no family history of diabetes, and parity≥3. The prevalence of T2D in non-obese (BMI < 30), good-oral health, and low-and high-PIR participants was significantly associated with the DII. A one-unit increase in DII accounted for an increment of 22% in the prevalence of T2D in non-obese patients (OR = 1.22, 95%CI: 1.08, 1.37), 17% in good-oral health participants (OR = 1.17, 95%CI: 1.07, 1.28), 17% in low-PIR participants (OR = 1.17, 95%CI: 1.05, 1.30), and 26% in high-PIR participants (OR = 1.26, 95%CI: 1.07, 1.48). The log-likelihood ratio test showed a significant interaction between DII and T2D among the obesity status, oral health, and PIR groups (all *p*for interaction <0.05). However, there were no significant differences among the other groups (all *p* for interaction >0.05). Further details are provided in Figure 2 and Supplementary Table S3.

Non-obese participants with good oral health were categorized into three groups based on their DII tertiles. The group with the highest DII had a 126 and 141% higher prevalence of T2D compared with the group with lowest DII (OR = 2.26, 95%CI: 1.49, 3.42 and OR = 2.41, 95% CI: 1.80, 3.22) amongnon-obese patients and those with good oral health, respectively (Table 3). In the adjusted model, the association was only slightly attenuated (OR = 1.96, 95%CI: 1.20, 3.19 and OR = 1.88, 95%CI: 1.31, 2.68, respectively). Additionally, there was a significant increasing trend in the occurrence of T2D across the DII tertiles for both non-obese and good oral health participants in the crude model (*p* for trend = 0.011, *p* for trend<0.011, respectively). Similar findings were observed in participants with low and high PIR. In the final model (Table 3), the group with the highest DII had a 76 and 163% higher prevalence of T2D compared with the group with thelowest DII (OR = 1.76, 95% CI: 1.14, 2.70 and OR = 2.63, 95%CI: 1.41, 4.91).

**Table 3:** Weighted multi regression analysis of the DII on type 2 diabetes stratified according to oral health, BMI, and PIR.

Population	DII	Crude model	Adjusted model
**Good oral health**	Continuous	1.26 (1.17, 1.35)	1.17 (1.07,1.28)
	Q1	Ref	Ref
	Q2	1.61 (1.23, 2.11)	1.40 (1, 1.97)
	Q3	2.41 (1.80,3.22)	1.88 (1.31, 2.68)
	*P* for trend	<0.001	<0.001
**Non obesity**	Continuous	1.26 (1.15, 1.39)	1.21 (1.07,1.36)
	Q1	Ref	Ref
	Q2	1.43 (0.96, 2.15)	1.27 (0.80, 2.03)
	Q3	2.26 (1.49, 3.42)	1.96 (1.20, 3.19)
	*P* for trend	<0.001	0.007
**Low PIR**	Continuous	1.12 (1.02,1.24)	1.17 (1.05,1.30)
	Q1	Ref	Ref
	Q2	1.09 (0.74,1.60)	1.17 (0.78,1.76)
	Q3	1.52 (1.01,2.29)	1.76 (1.14,2.70)
	*P* for trend	0.026	0.011
**High PIR**	Continuous	1.29 (1.15, 1.45)	1.26 (1.07, 1.49)
	Q1	Ref	Ref
	Q2	1.35 (0.85, 2.14)	1.26 (0.69,2.30)
	Q3	2.72 (1.66, 4.45)	2.63 (1.41, 4.91)
	*P* for trend	<0.001	0.005

Adjust forage, race, college education, marital status, poverty income ratio, physical activity, family history of diabetes, history of gestational diabetes, parity, BMI, oral health. DII, Dietary Inflammatory Index. BMI, body mass index. PIR, poverty income ratio.

Additionally, we examined the correlation between the DII and fasting glucose levels using fasting plasma glucose as an outcome variable. Regardless of whether DII was used as a continuous or trichotomous categorical variable, a positive association was found with fasting glucose levels (*β* = 0.83, 95%CI: 0.29, 1.37 and *β* = 2.98, 95% CI: 0.69, 5.27; p for trend = 0.011). However, the association between DII and fasting glucose was not significant when demographic information, BMI, and oral health were included as covariates (Table 4).

**Table 4:** Multi regression analysis of the association between DII and fasting plasma glucose.

	Fasting blood glucose (FPG, mg/dl)			
	Crude model		Adjusted model	
DII	*β* (95%Cl)	*P* value	*β* (95%Cl)	*P* value
Continuous	0.83 (0.29, 1.37)	0.003	0.19 (−0.33, 0.70)	0.477
Q1	Ref		Ref	
Q2	1.55 (−1.09,4.20)	0.247	0.04 (−2.54, 2.61)	0.978
Q3	2.98 (0.69, 5.27)	0.011	0.75 (−1.51, 3.02)	0.509
*P* for trend		0.011		0.515

Adjust for age, race, college education, poverty income ratio, family history of diabetes, history of gestational diabetes, parity. BMI, oral health. DII, Dietary Inflammatory Index. FPG, fasting plasma glucose.

## 4 Discussion

This study showed a direct link between DII and T2D in women in the United States. This association was confirmed using sensitivity and subgroup analyses.

Currently, the mechanisms of DII, diabetes, and insulin resistance are not completely understood (27). People with T2D often have mild inflammation that activates the immune system by producing pro-inflammatorycytokines (3). Diabetes causes ongoing inflammation due to factors such as high blood sugar, lipotoxicosis, and oxidative stress. Moreover, inflammation and oxidative stress worsen as diabetes and related conditions progress (23). The study is consistent with previous literature on the link between inflammation and diabetes: Zheng’s study found that higher dietary diversity scores and lower pro-inflammatory diets were associated with incident T2D in adults from the UK and US (28). In another study, the DII was found to be positively associated with fasting plasma glucose, fasting serum insulin, and the homeostatic model assessment of insulin resistance, and a more pro-inflammatory diet was associated with increased odds of insulin resistance and prediabetes (27). Dana E. Kingobserved a significant association between the severity of diabetes and DII scores in people with diabetes; a 1-point increase in the DII score was associated with a 43% (95%CI, 1.21, 1.68) increase in the odds of having an glycosylated hemoglobin above 9% (29). At the same time, certain dietary patterns may affect low-grade inflammation or body composition, thus influencing the incidence and development of some chronic diseases. For example, studies have shown that diets high in advanced glycation end products and antioxidants, such as the Mediterranean diet, may have a beneficial effect on health (30, 31).

It is clear that many chronic non-communicable diseases are the result of the accumulation of unhealthy lifestyles (32). There is no doubt that women’s health in the postnatal period is a critical period for the long-term health of mothers. Women are more motivated to engage in behavioral changes for their families to maintain their health, so we must take advantage of this “window”. This study aimed to examine the dietary habits of women with a reproductive history (excluding pregnant and lactating women) to investigate the association between DII and T2D.

Periodontitis is known to be a complication in diabetic patients (33). Diet is recognized as an important new modifiable factor that regulates the systemic inflammatory state (34), and there is a correlation between the inflammatory potential of diet and poor periodontal health (35). Many researches have confirmed that periodontal disease can act synergistically to amplify inflammatory and oxidative states, leading to an increase in local and systemic biomarkers (36). Convers5ely, oral infections and the local and systemic inflammatory responses they cause also can have a detrimental effect on blood glucose levels (37), moreover diabetes may counteract the role of anti-inflammatory diets in reducing periodontitis (38). Studies have shown that self-reported oral health status is associated with systemic comorbidities and has a similar correlation with periodontal disease (39), so the present study included oral health as an important covariate in the analyses.

Previous studies have demonstrated that periodontal disease is more prevalent among socially disadvantaged groups in the United States. These groups include low-income, uninsured, racial/ethnic minority, immigrant, or rural populations who have difficulty accessing high-quality oral health care and a greater likelihood of having poor oral health and high risk of chronic noncommunicable diseases (40). Consequently, these groups have become the focus of researchers’ attention. However we found a significant association between DII and T2DM among those with good oral health or normal PIR status interestingly. Given the limited sample size of participants in our study, our findings should be interpreted with caution and further research is needed in the future to explore the relationship between dietary inflammatory potential, oral health, and T2D.

Obesity is acknowledged as a significant public health issue and the primary risk factor for diabetes development (41). The rates of obesity and diabetes are increasing in parallel, resulting in higher mortality rates (41). Moreover, a higher DII score is linked to increased overweight/obesity risk (42). Notably, there is a transfer of obesity and diabetes across generations among women of childbearing age, prompting the development of strategies to enhance their health (43). However, less focus has been placed on the non-obese population. Other results in this study suggest that the pro-inflammatory effects of diet on T2DM may be particularly unfavorable in the non-obese population (BMI <30 kg/m^2^), which is an area that warrants further investigation. Similar results were obtained in a study by S. Galic, whose findings indicated a potentially stronger association between DII and T2DM risk in underweight or normal-weight participants, which was not significant when compared with overweight and obese participants (44). Another study indicated that there is a positive association between DII and the risk of IR in underweight and healthy weight adults (45). In a further study of postmenopausal Hispanic women, it was observed that obesity did not appear to modify the effect of the E-DII on the risk of incident diabetes (46). A recent study by Denova-Gutiérrez et al. (47) suggests that a pro-inflammatory diet may be associated with a higher likelihood of developing type 2 diabetes in adult Mexicans. The authors also observed an impact of the pro-inflammatory diet on body mass index, which was not associated with type 2 diabetes in participants with a body mass index <25 kg/m^2^ but was associated in participants with a body mass index ≥25 kg/m^2^ (44). In light of the current evidence, it seems that there is still much to be discovered regarding the potential benefits of reducing dietary inflammation in diabetes. While some studies have hinted at a link between dietary inflammation and diabetes, the evidence is still inconclusive and limited. This may be due to several factors, including a lack of large-scale studies, small sample sizes, differences in participant characteristics (e.g., gender and ethnic background), and variations in the parameters used to calculate the Dietary Inflammatory Index. It is clear that more research is needed to fully understand the relationship between dietary inflammation and diabetes. In particular, high-quality prospective studies and well-designed controlled trials could provide valuable insights.

The present study was based on a large, nationally representative survey, which allowed adjustment for multiple covariates and increased the statistical power of the results. Despite its strengths, we also acknowledge that this study has some limitations. First, as a cross-sectional study, the causal effect of DII and the risk of T2D in patients among women in the United States needs to be validated and extended in prospective studies. Second, T2D was defined based on a self-administered questionnaire without definitive quantitative indicators and failure to follow the American Diabetes Association criteria; however, the present study was a secondary analysis of a large population-based survey, in which diabetes prevalence was close to that in a previous report (48). Third, the DII was calculated from in-person 24-h recall data, which is inherently biased. In addition, we extracted the 24-h dietary information to represent the daily pattern, which may change over time. Fourth, due to the observational study design, our results will inevitably be affected by residual confounding due to unmeasured covariates. We constructed multivariable logistic regression models and performed subgroup and sensitivity analyses to control for the effects of potential confounders on the relationship between DII score and T2D. Fifth, the interaction between PIR in dII and T2DM was not analyzed in more detail. This will be investigated in more detail later. Finally, the NHANES data is from the United States population. Therefore, the results should be cautiously extrapolated to other populations in different countries. Thus, this study should be interpreted cautiously regarding the association between DII and T2D. More attention should be paid to the interaction effects of oral health and BMI on this association in the prevention and management of diabetes.

In conclusion, this study showed a significant positive association between DII and T2DM. Notably, the effect of dietary inflammation on T2DM was significant in non-obese, good oral health participants compared to obese, poor oral health participants. In addition, the relationship between DII and T2DM was sensitive to PIR.

The clinical significance of this study is multiple: firstly, it highlights the importance of dietary management in the female population with a reproductive history, recommending diets with lower DII. Secondly, the study suggests that non-obese participants with good oral health may benefit more from adopting a low inflammatory potential dietary pattern. Finally, the findings highlight the importance of tailoring dietary recommendations for specific populations (e.g., non-obese, good oral health participants) to prevent T2DM for long-term health promotion.

## Data Availability

The raw data supporting the conclusions of this article will be made available by the authors, without undue reservation.

## Footnotes

^1^ http://www.cdc.gov/nchs/nhanes.htm
