# Supplementary material for: Dietary inflammatory index and type 2 diabetes in US women: a cross-sectional analysis of the National Health and Nutrition Examination Survey, 2007–2018
Source: Front Nutr. 2024 Aug 14;11:1455521. doi: 10.3389/fnut.2024.1455521 (PMC11351284; doi:10.3389/fnut.2024.1455521)
Supplement: Supplementary file 1 [file Data_Sheet_1.docx]

Supplementary Material

Dietary Inflammatory Index and type 2 diabetes in US women: a crosssectional analysis of the National Health and Nutrition Examination Survey, 2007-2018

Tingyan Mo ^1^, Man Wei^2^, Jinyan Fu^1*^

*** Correspondence:** Jinyan Fu wenmail@hhu.edu.cn

# Supplementary Figures and Tables

## Supplementary Figures

**Supplementary Figure 1.** Association between dietary inflammatory index and type 2 diabetes


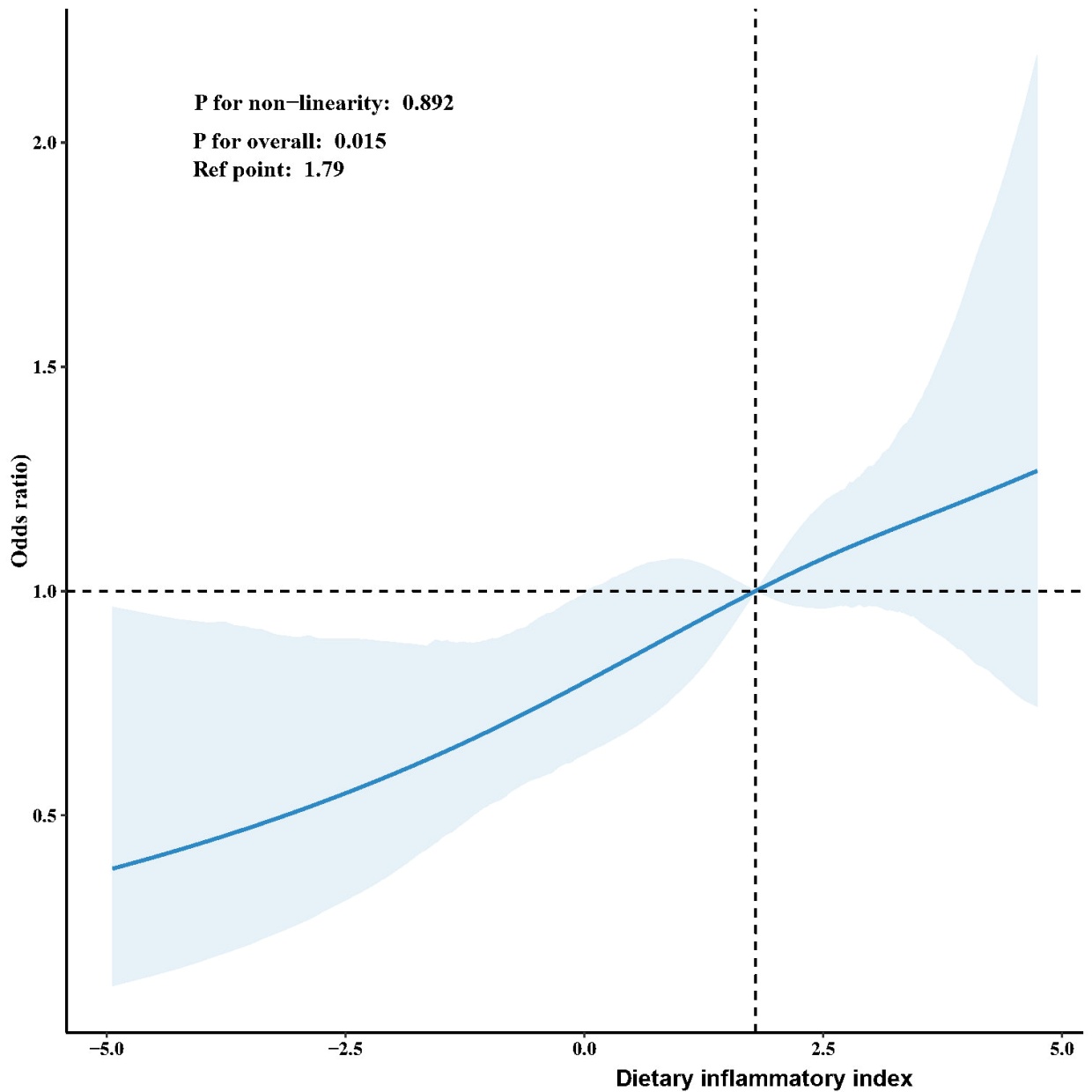


## Supplementary Tables

Supplementary Table 1. Baseline data for inclusion and exclusion

| **Charcateristic** | **Overall** | |
| --- | --- | --- |
|  | **Include，n=8394** | **Exclude，n=3823** |
| Age,mean±SD(years) | 53.722 (16.174) | 50.864 (17.255) |
| Race/ethnicity(%) |  |  |
| Non-Hispanic White | 3677.00 (43.81) | 1263.00 (33.04) |
| Others | 4717.00 (56.19) | 2560.00 (66.96) |
| College education(%) |  |  |
| No | 4175.00 (49.79) | 1882.00 (49.36) |
| Yes | 4211.00 (50.21) | 1931.00 (50.64) |
| Marital status(%) |  |  |
| Married or living with a partner | 4821.00 (57.46) | 2210.00 (57.88) |
| Living alone | 3569.00 (42.54) | 1608.00 (42.12) |
| Poverty income ratio,median,IQR | 1.920 [1.040, 3.770] | 1.790 [0.980, 3.430] |
| Tobacco use(%) |  |  |
| No | 5148.00 (61.35) | 2513.00 (65.79) |
| Yes | 3243.00 (38.65) | 1307.00 (34.21) |
| Alcohol use(%) |  |  |
| No | 3479.00 (41.49) | 1142.00 (29.89) |
| Yes | 4907.00 (58.51) | 2679.00 (70.11) |
| Physical activity,mean±SD(MET) | 2241.479 (4474.219) | 2728.017 (5361.305) |
| History of gestational diabetes(%) |  |  |
| No | 7726.00 (92.21) | 3489.00 (91.50) |
| Yes | 653.00 ( 7.79) | 324.00 ( 8.50) |
| Family history of diabetes(%) |  |  |
| No | 4508.00 (54.60) | 1957.00 (52.02) |
| Yes | 3748.00 (45.40) | 1805.00 (47.98) |
| Parity(%) |  |  |
| 1 or 2 | 4371.00 (52.07) | 1991.00 (52.08) |
| ≥3 | 4023.00 (47.93) | 1832.00 (47.92) |
| BMI,mean±SD(kg/m^2^) | 30.122 (7.393) | 29.982 (7.661) |

**Supplementary Table 1.** (Continued)

| **Charcateristic** | **Overall** | |
| --- | --- | --- |
|  | **Include，n=8394** | **Exclude，n=3823** |
| Oral health(%) |  |  |
| Excellent | 946.00 (11.66) | 361.00 ( 9.68) |
| Very good | 1512.00 (18.63) | 712.00 (19.09) |
| Good | 2796.00 (34.46) | 1251.00 (33.55) |
| Fair | 1814.00 (22.36) | 936.00 (25.10) |
| Poor | 1046.00 (12.89) | 469.00 (12.58) |
| WBC,mean±SD(10^9^/L) | 7.248 (2.302) | 7.400 (2.175) |
| Health insurance(%) |  |  |
| No | 1545.00 (18.41) | 681.00 (17.81) |
| Yes | 6849.00 (81.59) | 3142.00 (82.19) |
| Type 2 diabetes(~~%~~) |  |  |
| No | 7185.00 (85.60) | 3269.00 (85.64) |
| Yes | 1209.00 (14.40) | 548.00 (14.36) |

**Supplementary Table 2.** Characteristics of Survey Participants Included in Analysis (N = 59,647,927), National Health and Nutrition Examination Survey,2007-2018

| **Charcateristic** | **Dietary Inflammatory Index(DII)^c^** | | | | |
| --- | --- | --- | --- | --- | --- |
|  | **Overall** | **Q1** | **Q2** | **Q3** | **P value** |
|  | n=59647927.16 | n=22259546.76 | n=19655569.45 | n=17732810.96 |  |
| Type 2 diabetes(~~%~~) |  |  |  |  |  |
| No | 53192165.40 (89.18) | 20427617.11 (91.77) | 17579889.48 (89.44) | 15184658.81 (85.63) | <0.0001 |
| Yes | 6455761.76 (10.82) | 1831929.65 ( 8.23) | 2075679.96 (10.56) | 2548152.15 (14.37) |  |
| Age,mean±SD(years) | 53.01 (15.55) | 54.52 (14.30) | 52.05 (15.76) | 52.20 (16.63) | <0.0001 |
| Race/ethnicity(%) |  |  |  |  |  |
| Non-Hispanic White | 41321983.61 (69.28) | 16397811.66 (73.67) | 13270296.51 (67.51) | 11653875.43 (65.72) | <0.0001 |
| Others^a^ | 18325943.55 (30.72) | 5861735.09 (26.33) | 6385272.93 (32.49) | 6078935.53 (34.28) |  |
| College education(%) |  |  |  |  |  |
| No | 24746183.57 (41.50) | 6379180.78 (28.66) | 8814578.37 (44.88) | 9552424.42 (53.88) | <0.0001 |
| Yes | 34877750.33 (58.50) | 15875181.89 (71.34) | 10825140.24 (55.12) | 8177428.20 (46.12) |  |
| Marital status(%) |  |  |  |  |  |
| Married or living with a partner | 38625038.19 (64.78) | 15406838.07 (69.26) | 12808536.01 (65.17) | 10409664.11 (58.71) | <0.0001 |
| Living alone | 21003270.69 (35.22) | 6837697.62 (30.74) | 6845634.71 (34.83) | 7319938.35 (41.29) |  |
| Poverty income ratio,median,IQR | 2.72 [1.35, 4.86] | 3.65 [1.89, 5.00] | 2.67 [1.32, 4.63] | 1.95 [1.03, 3.65] | <0.0001 |
| Tobacco use(%) |  |  |  |  |  |
| No | 34807271.18 (58.39) | 13983074.59 (62.90) | 11912212.25 (60.63) | 8911984.35 (50.26) | <0.0001 |
| Yes | 24803466.47 (41.61) | 8248001.92 (37.10) | 7734637.94 (39.37) | 8820826.61 (49.74) |  |

**Supplementary Table 2.** (Continued)

| **Charcateristic** | **Dietary Inflammatory Index(DII)c** | | | | | |
| --- | --- | --- | --- | --- | --- | --- |
| **Charcateristic** | | **Overall** | **Q1** | **Q2** | **Q3** | **P value** |
|  |  | n=59647927.16 | n=22259546.76 | n=19655569.45 | n=17732810.96 |  |
| Alcohol use(%) | |  |  |  |  |  |
| No | | 19891199.82 (33.37) | 6451857.75 (29.01) | 6574400.01 (33.49) | 6864942.06 (38.73) | <0.0001 |
| Yes | | 39708970.51 (66.63) | 15790190.22 (70.99) | 13059274.00 (66.51) | 10859506.29 (61.27) |  |
| Physical activity,mean±SD(MET) | | 2307.79 (4327.24) | 2229.72 (3551.65) | 2145.56 (4327.08) | 2585.60(5128.17) | 0.0535 |
| History of gestational diabetes(%) | |  |  |  |  |  |
| No | | 54991531.00 (92.43) | 20516187.48 (92.33) | 18007259.87 (91.86) | 16468083.65 (93.20) | 0.3763 |
| Yes | | 4501958.75 ( 7.57) | 1703652.08 ( 7.67) | 1596473.88 ( 8.14) | 1201832.80 ( 6.80) |  |
| Family history of diabetes(%) | |  |  |  |  |  |
| No | | 34250340.46 (58.40) | 13725159.57 (62.51) | 10841257.37 (56.19) | 9683923.52 (55.67) | 0.0002 |
| Yes | | 24399673.49 (41.60) | 8232999.95 (37.49) | 8453949.33 (43.81) | 7712724.22 (44.33) |  |
| Parity^b^(%) | |  |  |  |  |  |
| 1 or 2 | | 35312512.29 (59.20) | 13978701.15 (62.80) | 11786120.59 (59.96) | 9547690.55 (53.84) | <0.0001 |
| ≥3 | | 24335414.87 (40.80) | 8280845.61 (37.20) | 7869448.86 (40.04) | 8185120.41 (46.16) |  |
| BMI,mean±SD(kg/m^2^) | | 29.54 (7.31) | 28.61 (6.99) | 29.92 (7.49) | 30.31 (7.39) | <0.0001 |
| Oral health(%) | |  |  |  |  |  |
| Poor | | 16635698.94 (28.53) | 4980838.78 (22.66) | 5602932.83 (29.20) | 6051927.33 (35.28) | <0.0001 |
| Good | | 41683902.44 (71.47) | 16995697.89 (77.34) | 13584186.60 (70.80) | 11104017.95 (64.72) |  |
| WBC, mean±SD(10^9^/L) | | 7.296 (2.259) | 7.005 (2.083) | 7.393 (2.226) | 7.555 (2.461) | <0.0001 |

**Supplementary Table 2.** (Continued)

| **Charcateristic** | **Dietary Inflammatory Index(DII)c** | | | | | |
| --- | --- | --- | --- | --- | --- | --- |
| **Charcateristic** | | **Overall** | **Q1** | **Q2** | **Q3** | **P value** |
|  |  | n=59647927.16 | n=22259546.76 | n=19655569.45 | n=17732810.96 |  |
| Health insurance(%) | |  |  |  |  |  |
| No | | 8386476.96 (14.06) | 2472828.59 (11.11) | 2837525.01 (14.44) | 3076123.36 (17.35) | 0.0001 |
| Yes | | 51261450.20 (85.94) | 19786718.17 (88.89) | 16818044.43 (85.56) | 14656687.60 (82.65) |  |

Abbreviations: DII, Dietary Inflammatory Index.MET, metabolic equivalent .BMI, body mass index.WBC,white blood cell.

^a^“Other” includes Mexican American, Other Hispanic, Non-Hispanic Black, Other Race - Including Multi-Racial.^b^Number of children. ^c^Data are presented as weighted numbers (weighted percentage) for categorical variables and mean (SD) for continuous variables.

Supplementary Table 3. Weighted ORs (95%CIs) of association between DII and Type2 diabetes in subgroups.

| **Subgroup** | **OR(95%CI)** | **P value** | **P for interaction** |
| --- | --- | --- | --- |
| **Age** |  |  | 0.237 |
| ＜60 | 1.09(0.98, 1.22) | 0.12 |  |
| ≥60 | 1.15(1.07, 1.25) | <0.001 |  |
| **Race/ethnicity** |  |  | 0.632 |
| Non-Hispanic White | 1.12(1.01, 1.24) | 0.04 |  |
| Others | 1.12(1.05, 1.19) | <0.001 |  |
| **College education** |  |  | 0.401 |
| No | 1.09(0.98, 1.22) | 0.12 |  |
| Yes | 1.14(1.03, 1.25) | 0.01 |  |
| **Marital status** |  |  | 0.632 |
| Married or living with a partner | 1.14(1.02, 1.28) | 0.02 |  |
| Living alone | 1.10(1.00, 1.20) | 0.04 |  |
| **Poverty income ratio** |  |  | 0.016 |
| ＜1.3 | 1.17(1.05, 1.30) | 0.004 |  |
| 1.3-3.5 | 1.02(0.94, 1.11) | 0.68 |  |
| ＞3.5 | 1.26(1.07, 1.48) | 0.01 |  |
| **physical activity** |  |  | 0.495 |
| Inactive | 1.08(0.99, 1.19) | 0.09 |  |
| Active | 1.16(1.03, 1.30) | 0.02 |  |
| **History of gestational diabetes** |  |  | 0.542 |
| No | 1.10(1.02, 1.20) | 0.02 |  |
| Yes | 1.19(0.99, 1.44) | 0.07 |  |
| **Family history of diabetes** |  |  | 0.927 |
| No | 1.11(0.98, 1.24) | 0.09 |  |
| Yes | 1.13(1.03, 1.23) | 0.01 |  |
| **Parity** |  |  | 0.09 |
| 1 or 2 | 1.21(1.07, 1.36) | 0.003 |  |
| ≥3 | 1.05(0.96, 1.14) | 0.32 |  |

**Supplementary Table 3.** (Continued)

| **Subgroup** | **OR(95%CI)** | **P value** | **P for interaction** |
| --- | --- | --- | --- |
| **Obesity** |  |  | 0.032 |
| No | 1.22(1.08, 1.37) | 0.002 |  |
| Yes | 1.06(0.97, 1.17) | 0.2 |  |
| **Oral health** |  |  | 0.017 |
| Poor | 1.04(0.95, 1.13) | 0.39 |  |
| Good | 1.17(1.07, 1.28) | 0.001 |  |

Ajust for age,race,college education,marital status,poverty income ratio, physical activity, family history of diabetes, history of gestational diabetes,parity, BMI,oral health
